# Design and Evaluation of Personalized Services to Foster Active Aging: The Experience of Technology Pre-Validation in Italian Pilots

**DOI:** 10.3390/s23020797

**Published:** 2023-01-10

**Authors:** Letizia Lorusso, Miran Mosmondor, Andrej Grguric, Lara Toccafondi, Grazia D’Onofrio, Sergio Russo, Jure Lampe, Tarmo Pihl, Nicolas Mayer, Gianna Vignani, Isabelle Lesterpt, Lucie Vaamonde, Francesco Giuliani, Manuele Bonaccorsi, Carlo La Viola, Erika Rovini, Filippo Cavallo, Laura Fiorini

**Affiliations:** 1 Innovation and Research Unit, IRCCSFondazione Casa Sollievo della Sofferenza, 71013 San Giovanni Rotondo, Foggia, Italy; 2 Ericsson Nikola Tesla d.d., Krapinska 45, 10002 Zagreb, Croatia; 3 Umana Persone Development & Research Social Enterprise, 58100 Grosseto, Italy; 4 Clinical Psychology Service, Health Department, IRCCSFondazione Casa Sollievo della Sofferenza, 71013 San Giovanni Rotondo, Foggia, Italy; 5 SenLab, 1000 Ljubljana, Slovenia; 6 Sentab, 10134 Tallin, Estonia; 7 Ascora GMBH, D-27777 Ganderkesee, Germany; 8 Gérontopòle Nouvelle-Aquitaine, 87069 Limoges, France; 9 Co-Robotics s.r.l., 56125 Capannoli, Pisa, Italy; 10 Department of Industrial Engineering, University of Florence, 50139 Firenze, Italy

**Keywords:** pre-validation, technological personalized assistive service, older adults, active aging, pilots

## Abstract

Assistive devices could promote independent living and support the active and healthy aging of an older population; however, several factors can badly influence the long-term use of new technologies. In this context, this paper presents a two-step methodology called “pre-validation” that aims to identify the factors that can bias the use of new services, thus minimizing the risk of an unsuccessful longer trial. The proposed pre-validation methodology is composed of two main phases that aim to assess the usability and the reliability of the technology assessed in a laboratory environment and the usability, acceptability, user experience, and reliability of the technology in real environments. The tested services include the socialization scenario, in which older adults are better connected to the community via technological solutions (i.e., socialization applications), and the monitoring scenario, which allows for the introduction of timely interventions (technologies involved include environmental monitoring sensors, a telepresence robot, wearable sensors, and a personalized dashboard). The obtained results underline an acceptable usability level (average System Usability Scale score > 65) for the tested technologies (i.e., socialization applications and a telepresence robot). Phase Two also underlines the good acceptability, user experience, and usability of the tested services. The statistical analysis underlines a correlation between the stress related to the use of technology, digital skills, and intention of use, among other factors. Qualitative feedback also remarks on a correlation between older adults with low digital skills and an anxiety about using technology. Positive correlation indexes were highlighted between the trust and usability scores. Eventually, future long-term trials with assistive technology should rely on motivated caregivers, be founded on a strong recruitment process, and should reassure older adults—especially the ones with low digital literacy—about the use of technology by proposing personalized training and mentoring, if necessary, to increase the trust.

## 1. Introduction

The World Health Organization (WHO) [1] estimates that, by 2050, the global population aged ≥ 60 years will increase substantially. This growth is a matter of concern not only numerically but also for the frailty of this population [2]. Despite increased longevity and life expectance, aging leads to a lack of autonomy, cognitive impairment, isolation due to increasing loneliness, and diseases of different severity degrees. Today, technology represents one of the solutions to overcome this process. The concept of “aging in place” has been pointed out in many works [3,4]. As defined by Hechinger et al. [4], “*aging in place is living in one’s own home as long as possible while maintaining social and care networks and autonomy in deciding the type of assistance*”. However, as is highlighted by this work, the existing literature is fragmented and lacking in effective solutions. Currently, the most important challenge is to create technologies that meet the real needs of older adults, proving their impact on the assistance care path. Recent literature has shown that information- and communication-technology (ICT)-based solutions can be a suitable way to promote health among older adults [3,5,6], with special attention paid to those who are living in a rural context [5]. It has been observed that the use of wearable and environmental devices, in combination with appropriate activities, applications, and feedback [7], can improve the promotion of healthy habits [8,9]. Moreover, the caregiving relationship is crucial in promoting the health and well-being of older adults [10]. However, future studies should be tailored to prove the long-term impact of those technologies in real life, as most related works have focused more on short-term interaction (interactions of less than 6 months). Over the last year, several research works have attempted to assess the acceptability and usability of digital technologies among older adults [11,12,13]. Recently, Choukou et al. [11], in their scoping review, remarked that other stakeholders such as caregivers and professionals should be included in the evaluation of assistive devices, Indeed, less than 50% of identified studies evaluated acceptance in more than one population. At the same time, different works have attempted to define a methodology [3,4,14,15,16]. In particular, the LIFE methodology [17] underlines the necessity of conducting in-field tests before the long trial in order to fine-tune the systems, and Choukou et al. [11] remarked on the necessity of adopting a mixed-method research strategy, investigating usability, efficiency, and effectiveness beginning in the early stages of the project.

In this context [18], this paper presents a two-phase methodology—namely, pre-validation—that aims to pre-test the technology and the scenarios, involving different user groups and thus preventing the risk of participant drop-out, favoring compliance during the deployment phase [19]. The methodology and the evaluation protocol follow the literature guidelines identified in the recent literature findings. Particularly, Phase 1 aims to assess the usability and the technological reliability after a brief interaction (around 1 h) in which the participants (i.e., older adults and informal and formal caregivers) are asked to test the technologies, focusing on all possible weaknesses. The aim of Phase 2 is to assess the potential changes in usability [20], acceptability [21], user experience (including efficiency), and technological reliability after interactions lasting for approximately one month, in which the older adults test the technologies at home under “real-life conditions perspectives” [3]. Additionally, this study also measures the training and stress related to using the technology. At the beginning of the study, the participants are trained in the use of the technology to introduce the use of technologies as a guidance for older adults and their informal caregivers. It is important to quantify the training session to evaluate any factors that could affect the user experience [22]. The use of a standardized test that measured the perceived stress is important for avoiding the risk that stress can indirectly influence the use and the motivation to use [13]. Indeed, thanks to the prolonged use of the system during Phase 2, we can observe—and investigate—the factors that can potentially affect acceptance in real environments, minimizing the risk of failure in the long trial [23,24]. The pre-validation setting is based on mixed-method research and the use of standardized questionnaires, as needed and suggested in two papers by Choukou et al. [11]. Another strength of our methodology is the interdisciplinary team, in which mutual interactions with developers can overcome critical issues and provide immediate solutions [24,25]. In addition to evaluating the usability, acceptability, user experience, and the technical reliability of the proposed services, we also investigate the following research questions (RQs):

**RQ1:**  How do the acceptability and user experience affect usability?

Acceptability and usability are domains that mutually influence each other. In particular, trust and intention of use can be correlated with usability; if a participant does not trust the system, she/he will never use it, even if the relationship between these constructs can change according to the application context [26]. The usability and acceptability are assessed by the system usability scale (SUS) [20] and the Almere Model Questionnaire (AMQ) [21], respectively, whereas the user experience questionnaire is used to assess the user experience [27].

**RQ2:**  How does a proper training session affect usability and acceptability?

Older adults have low digital skills and are not accustomed to using technology in their life. Therefore, training represents one important pillar that can affect the use of the system in daily life [28,29]. In this study, we aim to investigate the correlation between the usability/acceptability score and a proper training session. We expect to find a positive correlation between these scores. We use the Training Evaluation Inventory (TEI) to evaluate the training sessions [30].

**RQ3:**  How may the stress related to the use of technology affect the acceptability and the intention to use the technology? How does it differ among the population?

Due to low digital literacy, participants (i.e., older adults and informal and formal caregivers) can be under stress when introduced to technology, which consequently affects the acceptance of the Pharaon system. To evaluate the increase or decrease of stress related to the use of technology during the same period, an adapted version of the Perceived Stress Scale (PSS) (see Appendix A) test is used. This score was correlated with the AMQ questionnaire.

## 2. Italian Pilots Architecture

The proposed services were developed in the context of the Pilots for Healthy and Active Ageing (Pharaon) project, which aims to the implement and evaluate six Large Scale Pilots (LSPs) in five EU countries. In particular, the Italian pilot [18] will deploy personalized health management, socialization, and inclusion support scenarios. This chapter will provide details on the Italian use cases and scenarios, including the implemented functionality, delivering an overview of how the Italian pilot is integrated into the Pharaon ecosystem. Additionally, this chapter provides details on how the Pharaon Reference Architecture was conceived and how the Italian System (based on the Pharaon Concrete Architecture for Italian Pilots) was implemented.

### 2.1. Use Cases and Scenarios

According to a needs analysis [31], two services were outlined. These were “health management and monitoring” and “socialization and inclusion support” scenarios. The health management and monitoring scenario required the monitoring of the older adults’ home environments, employing movement sensors and humidity and/or temperature sensors. The physical activity monitoring was measured using a smartwatch (Table 1). Regarding the socialization and stimulation service, our group proposed a social network platform with selected games and exercises to stimulate cognition and memory (Table 2). The platform allowed the possibility for an older adult to stay in touch with their own caregiver, family and friends, or a health professional. A list of the implemented functionalities is reported in Table 1 and Table 2.

### 2.2. Italian Pilot Sites as AAL Sub-Ecosystems

**Figure 1:**
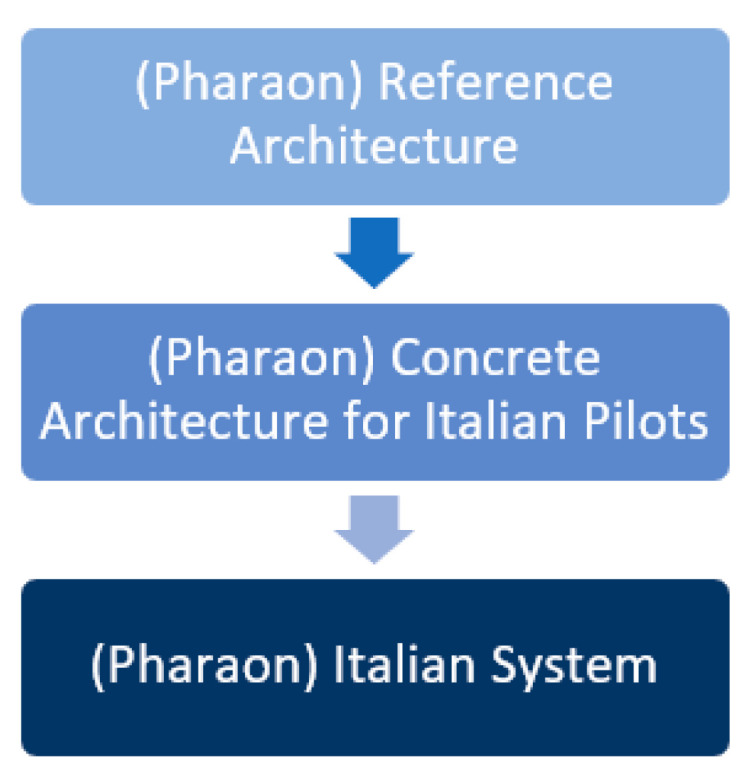
Relation between Pharaon Italian System, Pharaon Concrete Architecture and Pharaon Reference Architecture.

The [32] Pharaon ecosystem is envisioned as a Meta-AAL ecosystem with the coordination and centralization of the common aspects of all Pharaon pilots on one side, while allowing for federation and decentralized decision-making with autonomy specific to each Pharaon pilot. From the perspective of the Italian pilot, both Italian pilot sites were considered to be a socio-technical, AAL ecosystem at the meso level, while the Pharaon ecosystem is an AAL ecosystem at the macro level. For the definition of the macro and meso levels, we refer to the definitions from Poh et al. [33], who, following a systematic literature survey, identified the macro-level regulations as operational authorization, care quality assessment, and infrastructural requirements, and the meso-level regulations as operational management, staff management and distribution, service provision, care monitoring, and crisis management. Regarding the Italian System, we referred to the Pharaon technical subsystem for the technical realization and delivery of Italian pilot scenarios and use cases (Figure 1). Such a technical subsystem is supported by the social subsystem, fed with input from the (formal or informal) caregivers and other relevant stakeholders.

### 2.3. The Italian System in Pharaon Architecture

**Figure 2:**
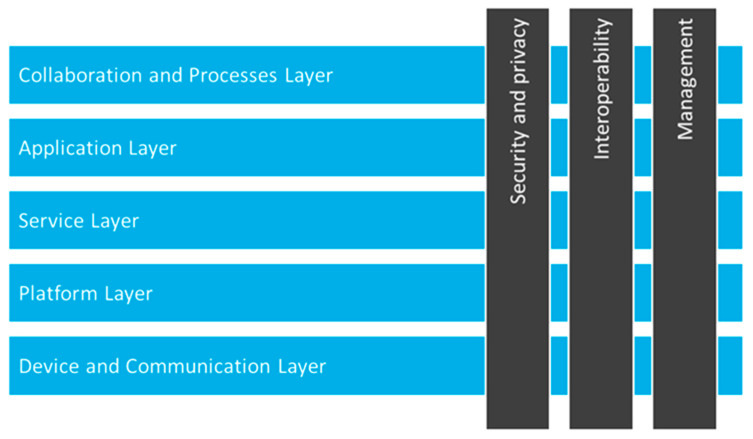
Pharaon’s high-level reference–logical architecture view.

Pharaon’s reference-architecture-related work was analyzed in more detail and compared with six other eHealth and AAL (Ambient/Active Assisted Living) projects. It was presented in [32]. Pharaon reference architecture is described using different architectural perspectives, from conceptual, logical, communication, informational, process, and system views. Pharaon’s logical view is a technology-agnostic view of the functions necessary to form a system for the realization of a set of Pharaon use cases. Pharaon’s high-level reference–logical architecture view is shown in the following figure (Figure 2). The horizontal (logical/functional) layers in Pharaon are *The Device and Communication layer, The Platform layer, The Service layer, The Application layer, and The Collaboration and Processes layer*. Aside from the horizontal functional layers, the logical view also defined cross-cutting functions (cross-domain functions) such as security and privacy, which were already considered in the initial design phase. More details regarding the Pharaon conceptual model, reference–logical architectural view, and the Pharaon ecosystem can be found in [34].

Scenarios and use cases were not realized by the implementation of a new system from scratch but by the customization and integration of existing devices, platforms, tools, and services provided by different partners of the project. Thus, the next step toward system realization was the mapping of a common, architectural, functional blueprint of selected partner technologies. The criteria for the selection of specific technologies to be used in each pilot were not only purely functional, in terms of whether certain technology implementations could be customized to support certain required functionalities, but also included operational and practical aspects. The operational criteria refer to the ability of a given technology to be deployed in a production/pilot environment and be supported and maintained by a technical partner during trials on the given pilot site.

The technology selection and mapping process resulted in the selection of the following technologies for use in the Italian pilots:

- *Sentab technology* (https://www.sentab.com/, accessed on 16 November 2022) is an end-to-end solution developed by the company of the same name for providing entertainment, social interaction, and monitoring for older adults and their families. It connects seniors with their caregivers and relatives seamlessly over TV and tablet interfaces for seniors and web and mobile interfaces for caregivers, providing, amongst others, video calling and media-sharing features. Sentab was used on the TV in the Tuscany pilot and on the tablet version in the Apulia pilot, according to the guidelines and feedback received during the needs analysis [31]. SENTAB also included the Vanilla web-based application, which was used by the caregivers to talk to and socialize with end-users.
- *Discovery Dashboard*, by Ascora (ASC), is a solution that provides a user interface (web dashboard) through which formal and informal caregivers can monitor collected and processed data from different environmental and wearable sensors. It also provides user profile management and user environment configuration features.
- *SmartHabits Platform* [35], by Ericsson Nikola Tesla d.d. (ENT), is part of an intelligent, privacy-aware home-care assistance solution that is used for data processing and uses machine-learning technology to detect anomalies (unusual values in sensor data and outliers).
- *IoTool* (https://iotool.io/, accessed on 16 November 2022), by Senlab, is primarily an IoT platform that helps connect IoT devices (sensors and robots) through a flexible and open extensions system via any interface to a smartphone, microcontroller, or directly to the IoTool servers in the edge or cloud. The collected data is encrypted, stored, processed, and can be sent to external systems for further processing.
- *The Ohmni Telepresence Robot* (https://ohmnilabs.com/products/ohmni-telepresence-robot/, accessed on 16 November 2022), is a third-party robotic solution that provides a telepresence service.
- Other third-party technologies, such as commercial environmental sensors and smartwatches.

**Figure 3:**
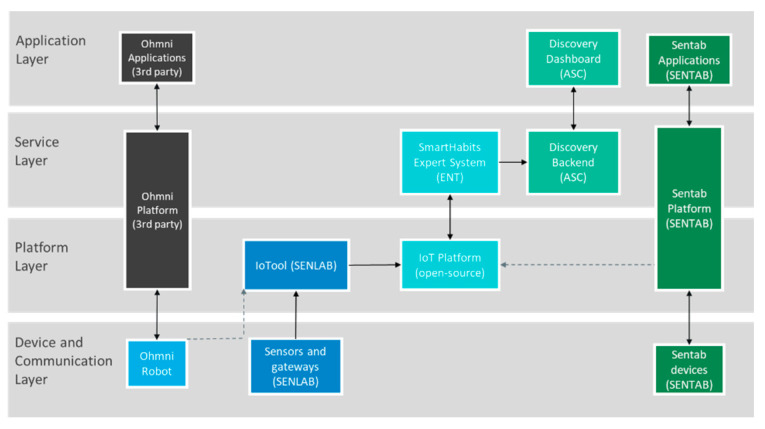
High-level system view of the Italian pilot.

The mapping between the high-level technologies’ building blocks (system view) and the Pharaon layered model is presented in Figure 3.

### 2.4. Italian System Implementation

**Figure 4:**
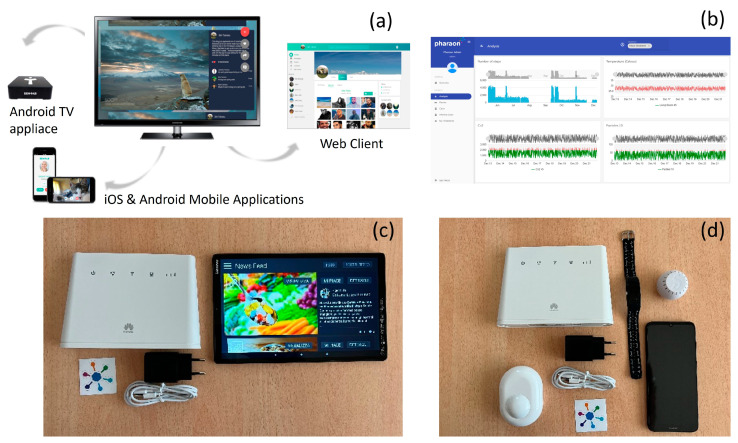
The technologies used were (**a**) the SENTAB system; (**b**) the Discovery Dashboard, an example of the data showed on the interface; (**c**) for the health management and monitoring scenario, the gateway or smartphone (if the older adult did not have one) were used to connect the smartwatch, the PIR movement, and humidity and temperature sensors; and (**d**) for the monitoring socialization and inclusion support, the gateway and the tablet were used with the SENTAB application installed on the tablet.

When discussing integration flows in the context of the Pharaon project we are referring to flows of data between technologies from different partners that were used for the implementation of certain scenarios. The socialization scenario was realized end-to-end by using technology from a single technology provider: Sentab. *Sentab* (Figure 4a) uses interoperable Native Client Applications across Android apps (targeting both the Android TV interface on the Android TV box, but also native Android apps for tablet and phone usage scenarios), iOS, and web applications. The backend solution is based on Enterprise Java on Jetty, open source RabbitMQ, and Redis dockers. Information is stored in a MYSQL database on Ubuntu servers. For WebRTC video calling, the solution uses Open-Source C Language Project in the TURN server, NodeJS for the signaling server, and Nginx for the reverse proxy server. The Content Delivery Network (CDN) is built on Amazon CloudFront and S3. The public services can be accessed via REST (REpresentational State Transfer) APIs using JSON data interchange formats. APIs are secured by JWT (JSON Web Tokens) and TLS (Transport Layer Security).

**Figure 5:**
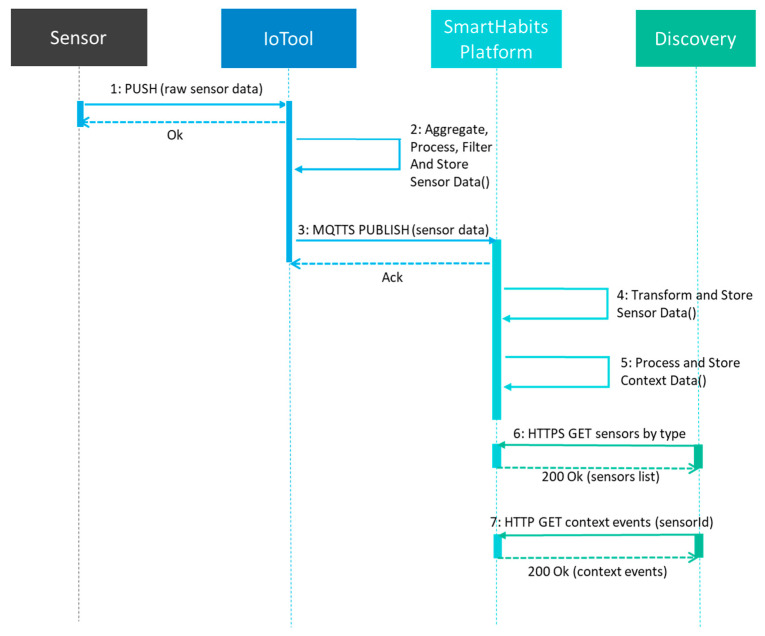
The high-level sensor data flow of the monitoring service.

The monitoring scenario, on the other hand, was realized by the integration of technologies from several different technology providers; namely *IoTool*, *SmartHabits Platform*, and *Discovery*. The high-level sensor data flow between different technologies in the monitoring scenario is presented in Figure 5.

There were two categories of sensor devices used in the monitoring scenario in pre-validation: well-being and environmental sensors. Although *IoTool* supports the integration of more than one hundred different devices, a particular device choice was made not only based on technical integration capabilities but also on other aspects, such as budget constraints and market availability (considering that commercial third-party devices were used). *IoTool* is an IoT solution incorporating a client, a dashboard, a gateway, and a Cloud. A smartphone or other device, such as a microcontroller, is a data collector and optionally a dashboard, controller, and a gateway to synchronize data to the Cloud. The collected data is encrypted, stored, displayed, processed, and synchronized to the Cloud (IoTool servers, self-deployed servers, or other IoT platforms, such as IBM Watson). The IoTool technology stack consists of Java/SQLite on the smartphone, Kubernetes/Docker with a PostgreSQL database, and a Mosquitto MQTT broker and client, node, node-red, and PHP.

The *SmartHabits Platform* is based on microservice architecture, providing great flexibility for responding to the needs of different scenarios. It is a Java enterprise solution based on the Spring Framework and other open-source technologies. Time-series sensor events, as recorded observations correlated to the time received from *IoTool*, and context events are stored in the MongoDB databases, while other contextual and configuration information are stored in the MySQL databases. The services expose their functionalities primarily via REST (REpresentational State Transfer) APIs use lightweight JSON (JavaScript Object Notation) as a data interchange format. All APIs are secured by JWT (JSON Web Tokens) and TLS (Transport Layer Security). Apart from using (operating-system- and programming-language-agnostic) APIs, the AMQP (Advanced Message Queuing Protocol) is also used for the intra-platform components’ communications. More technical details regarding the SmartHabits Platform in the scope of the Pharaon project have already been described in our previous work [34].

*Discovery* is based on a microservice architecture, making it more flexible regarding development languages and for the separation of concerns. Additionally, it is possible to scale it horizontally and downtime is less of an issue, given that at least one backup service is running for every microservice. The microservices are realized with different technologies, such as PHP, ASP.NET Core, Angular, and NodeJS/TypeScript. Information is pulled via a scheduled leecher service, which transforms the information into a format compatible with the analyzer component. These leechers are small services which can be created for every new source and can also be started externally if needed. The analyzer service creates knowledge out of this information and provides it to the API. This API is integrated into a DSS (Decision support System) called Discover UI, which has several methods (diagram types, etc.) of displaying data. Discovery UI is integrated in a profile-based component which organizes user profiles and handles authentication and the authorization of different boards of Discovery UI with the help of KeyCloak. Information is stored in mondoDB, and all APIs follow the OpenAPI standard and are secured by JWT and TLS (Figure 4b).

From a security perspective, each provider is responsible for properly securing their solution. The data exchange between these platforms has been secured by using Secure Sockets Layer (SSL) encryption, Application Programming Interface (API) tokens, and Internet Protocol (IP) address whitelisting mechanisms.

Activities, experiences, and lessons learned in the integration and deployment of technology in Pharaon have been described in [34]. As was mentioned in the article, achieving technical (syntactic) interoperability between these technologies, which includes the agreements between the data formats, protocols, adaptation of interfaces, and APIs, was just one segment of the overall technical and supporting activities necessary to achieve end-to-end integration. Despite the simple data flow presented, each technology block is a complex system, usually running microservices in a multi-cloud environment and using a vast number of additional components such as proxy servers, firewalls, load balancers, message brokers, cache servers, Structured Query Language (SQL) and NoSQL (not only SQL) databases, and event streaming platforms.

## 3. Pre-Validation Methodology of the Italian System

In this chapter, we will introduce the two-phase methodology (called pre-validation) that we used to assess the usability, acceptability, technological reliability, and user experience of the technologies presented in the previous chapter that were integrated to deliver the services outlined in Section 2.

### 3.1. Methodology

**Figure 6:**
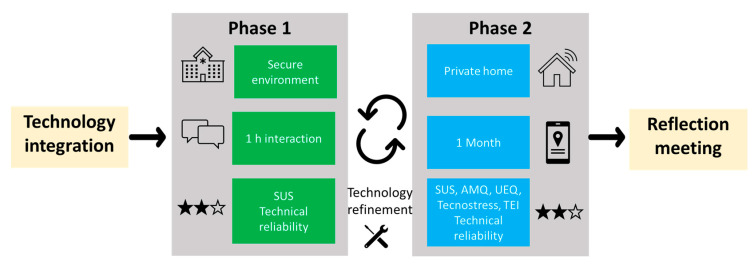
Highlights of the pre-validation phases: the different experimental settings, the duration of the interaction, and the evaluation framework. Phase 1 aimed to evaluate the stand-alone technology, whereas the Phase 2 aimed to evaluate the integrated services.

The proposed evaluation methodology was based on two main phases, Phase 1 and Phase 2 (Figure 6), that differ in timing, location, and evaluation protocol. The objective of Phase 1 was to assess the usability and the technical reliability of the stand-alone technology, whereas Phase 2 aimed to assess the usability, acceptability, and technological reliability of the integrated services for a prolonged time in real environments. In between the two phases, the technologies were updated to solve bugs and improve usability.

**Figure 7:**
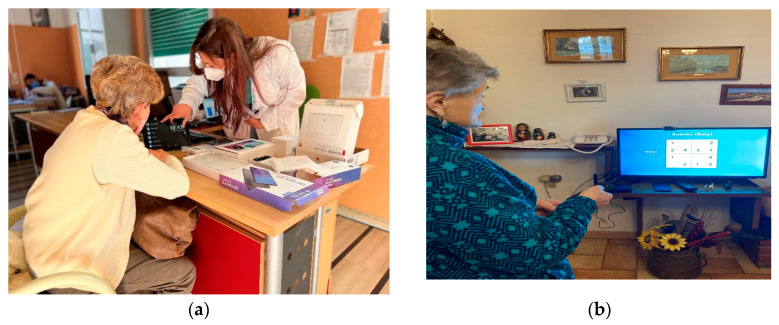
Pre-validation in Phase 2: (**a**) the figure shows how a training session developed during Phase 2 in Apulia. The trainer is showing the SENTAB application on the tablet; (**b**) In this figure, we can see an older adult in Tuscany using a Sudoku puzzle with a 2 × 2 grid (a stimulation exercise tool) on the SENTAB Application. The application is installed on her TV at home.

**Phase 1** of the pre-validation was performed through one-to-one private interactions in a controlled environment (i.e., a residential facility in Tuscany and a hospital in Apulia) (Figure 7a). Each interaction lasted around 1 h. In the beginning, the participants were welcomed and then the technology and the services were introduced and shown. The participants were then asked to actively interact with the technology. Two trained facilitators were requested to facilitate the interactions, address the questionnaire, track the technical bugs, and annotate events, comments, and feedback during the interactions. To harmonize the data collection, the methods, and the role of the facilitators during the test session, a reference manual was prepared.

**Phase 2** of the pre-validation was performed by asking the participants to use the services (Table 3) in a free manner for one month. The applied methodology envisaged two separate sessions with recruited older adults and their associated informal caregivers (e.g., a child). The objectives of Session I were to introduce the study, acquire informed consent, and address the cognitive and quality of life questionnaire. The objectives of session II were to train the users in the systems and to address the questionnaires (see Appendix A). Similar to Phase I, a manual was delivered to users as a support tool during the testing Phase. At the Tuscany pilot site, Session II was performed in private homes, followed by the installation of the sensor. In Apulia, Session II was performed at the hospital where the users were recruited. At the end of Session II, the users were asked to use the technology in their daily life (Figure 7b). If they had had problems, they were able to contact the facilitators. The selected questionnaires were addressed at the end of the trial. According to the remarks collected at the end of Phase I, the stress related to the use of the technology was also assessed due to the poor digital literacy of the recruited participants.

At the end of Phase 2, to collect feedback and lessons learned from the proposed two-phase methodology, an interactive reflection meeting was organized involving the pilots’ facilitators and technical developers. In the first part of the meeting, every single participant was asked to provide evidence of the things that worked well (or did not work well) regarding services, technologies, and methodologies (i.e., training, installation, and timing activities). There was then a preliminary discussion, and the notes/actions were grouped, discussed, and prioritized according to their feasibility and importance. The MURAL software^®^ was used to facilitate data collection and remote active collaboration between partners. Two facilitators were present in the role of facilitating interactive parts and taking notes.

### 3.2. Evaluation Framework

The technologies and services were evaluated using a multidisciplinary evaluation framework that included qualitative and quantitative metrics for assessing usability, acceptability, user experience, and technological reliability. In particular,, the following tools were used:

- The usability was assessed using the System Usability Scale (SUS) questionnaire [20]. The selected test was the ten-item questionnaire described in Brooke [20]. The score of this test is between 0 and 100, measured by a Likert scale (from one to five). The SUS questionnaire is capable of acquiring a subjective assessment of the usability. A value below 68 was not considered acceptable. Nevertheless, a score between 50 and 68 is considered a marginal score, and does not mean strictly non-acceptable [36]. In this phase, we expected the resulting score to be higher than or equal to 68. If not, improvements needed to be made by the technology providers.
- The acceptance was assessed using the Almere Model Questionnaire (AMQ) [21], which made 39 items available at [37]. The questionnaire used in this study was based on the original test by Heerink and adapted for the Pharaon technologies. The constructs of Perceived Sociability (PS) and Social Presence (SP) were omitted because they were out of the scope of this work. The negative items 1,2,3,4, and 36 had a reverse score. The full list of items and constructs used are reported in Appendix A, Table A1. The AMQ was designed with the aim of being applicable to vulnerable people such as older adults [38].
- The training evaluation was performed using the Training Evaluation Inventory (TEI) developed by Ritzmann et al. [30]. For this study, we chose the first seventeen items (the items and the respective Italian translations are displayed in Appendix A, Table A3).
- The term “*technostress*” had been defined in previous research by Brod [39] and was measured in different research contexts. Fischer et al. developed a new tool to assess the digital stress perceptions [40], but the most commonly used test is the Perceived Stress Scale (PSS). The aim of the PSS is to quantify the perceived stress related to the use of technology [41,42], testing the differences in perceived stress. In most cases, the test has been administered at two times: at the beginning and end of a period of using technological solutions (web-based technologies, smartphones, applications, etc.) and with different participant groups [43,44]. In this study, the stress related to technology was assessed by the Perceived Stress Scale (PSS) test [45], adapted as is shown in Appendix A, Table A2. In this paper, the test was renamed as the Technostress test. The score used was the same as in the PSS, with 0 = Never; 1 = Almost Never; 2 = Sometimes; 3 = Fairly Often; and 4 = Very Often. The individual score ranges from 0 to 40, and higher scores indicate higher levels of perceived stress. Scores ranging from 0 to 13 would be considered low stress; 14 to 26 would be considered moderate stress; and 27 to 40 would be considered high perceived stress. For the positive items, 4,5,7, and 8 had reverse scoring. The original PSS questions referred to a time period of one month; in our case, the period was modified according to the timeline reported in Table 4.
- The user experience was assessed through the User Experience Questionnaire [46,47] that assesses attractiveness, perspicuity, efficiency, dependability, stimulation, and novelty.
- The reliability of the technology was assessed by asking the facilitators to keep track of malfunctioning using the project’s issue board (hosted on a private GitLab repository), assigning a “priority” label to classify the malfunction (i.e., high, medium, and low risk) according to the impact it had on the pilot. Additionally, the facilitators were requested to use a diary to annotate all the qualitative feedback.

Following the guidelines presented by Broekhuis at al. [48], all the questionnaires were coupled with the “thinking aloud” method. Indeed, at the time of the questionnaire administration, the participants were asked to freely talk about their thoughts, which were then be annotated and used to explain/integrate the quantitative results. Table 4 reports the timing of questionnaire administration (i.e., Phase 1; Phase 2 at the beginning (T0); Phase 2 at the end of the testing phase (TF)).

### 3.3. Participants

In Tuscany, the participants were recruited by the Umana Persone Social Enterprise R&D Network (UP). In Apulia, they were recruited at the Casa Sollievo della Sofferenza (CSS) Foundation research hospital, composed of different clinical units, and at the Casa Padre Pio residence for older adults. At the beginning of recruitment, some sociodemographic information (i.e., digital skills, educational level, age, and gender) was collected for all target groups (Older adult, OA; Informal Caregiver, IC; or Formal Caregiver, FC). The inclusion and exclusion criteria were the same in Phase 1 and Phase 2. The inclusion criteria for older adults were:

- Aged ≥60 years old;
- Having the ability to provide informed consent or the availability of relatives or a legal guardian in the case of severely demented patients (a MMSE score ≥ 18 was requested);
- A frailty score from two (well) to six (moderately frail) on the Canadian Scale [49].

The exclusion criteria, present only for older adults, were:

- The presence of severe cognitive impairments;
- Other causes that can cause memory impairments or difficulties with engagement.

The inclusion criteria for the formal/professional caregivers were:

- Motivation;
- Basic digital skills.

There were neither inclusion nor exclusion criteria for informal caregivers.

### 3.4. Ethics Compliance

In Tuscany, the pre-validation was approved by the Ethical Committee of Azienda USL Toscana Sud-Est on 22/07/2021 (prot. 2021/000227). In the Apulian pilot, the pre-validation Phase 1 and Phase 2 ethics requests were approved by the ethical committee on the 14th of June 2021 with the number protocol 1669/01 DG. The ethics committee requests and approvals have been compliant with the EU GDPR and national recommendations. The informed consent form was signed by all users involved.

### 3.5. Data Analysis

Questionnaire reliability was assessed using Cronbach’s alpha analysis as a measure of internal consistency. Cronbach’s alpha ranges from 0 to 1:0.7 was considered the minimal acceptable value, otherwise one or more items were deleted.

Regarding the SUS score, the descriptive statistics were computed following the guidelines in [20]. The same values were computed for the Technostress, AMQ, and the TEI values. For UEQ, data analysis provided by https://www.ueq-online.org/ (accessed on 16 November 2022) was used. First, to assess the normality of the distribution, the Shapiro–Wilk normality test was computed. To investigate some differences in acceptability and usability between related samples, the Wilcoxon rank sum test was used, or the t-test was used if the variables were not normal or normally distributed, respectively. To show any differences among the three categories of users, the Kruskal–Wallis test was used for independent samples due to the small sample size. To investigate the RQs, the Pearson correlation coefficient (R_p_) was calculated only if both variables were individually normally distributed; otherwise, Spearman’s rank correlation coefficient (R_s_) was computed. In all cases, a significant *p*-value was held when the type 1 error rate was smaller than 0.05. The statistical analysis was performed with SAS^®^ OnDemand for Academics. The small sample size has not allowed us to carry out further statistical analysis. Considering the qualitative feedback, the diaries collected from caregivers and the oral feedback collected during the questionnaire administration were read, transcribed, and analyzed to corroborate the quantitative analysis.

## 4. Results

A total of 27 persons were recruited within Phase 1 and 30 persons were recruited for Phase 2, bringing the total to 57 subjects. Unfortunately, three older adults dropped out at the beginning of Phase 2, so we included only 27 participants in Phase 2 (Table 5). The older adults recruited had an MMSE score of mean ± SD = 25.5 ± 3.7 and a Rockwood frailty score of mean ± SD = 2.7 ± 1.0. Eleven participants were involved in the final reflection meeting.

### 4.1. Phase 1 Results

A description of the participant cohort is reported in Table 6. The educational level was assessed through the International Standard Classification of Education (ISCED), 2011. The nine educational levels were clustered into three categories: Group One for ISCED categories 0, 1, and 2; Group Two for ISCED categories 3, 4, and 5 and Group Three for ISCED categories 6, 7, and 8. Regarding the digital skills of the participants, it was considered a level one if the participants had no skill or had only basic skills; a level two if they had intermediate experience; and a level three if their digital skills were advanced or excellent. A statistical analysis underlined that the three groups differed only in age, as was expected for the participant group involved in the study.

According to the SUS results (Table 7) for the Discovery Dashboard, we obtained a good usability score for both informal and formal caregivers. However, we had a low usability score for the older adult cohort. These results were not sufficient to pre-validate this technology. This could be explained by some oral feedback collected, as older adults had difficulties understanding and viewing the data graphs due to visual problems. According to this feedback, the pilot site decided to adopt Discovery only for the informal and formal caregivers for the next phases. The SENTAB scenarios succeeded (Table 7), especially the video call, in which the older adults were happy to be able to see their parents on a “big” screen. The SUS results, however, were too low for the adoption of the technology, which could be explained by the need to use a remote control, which was difficult for the older adults. The Ohmni robot was pre-validated in Tuscany only with older adults and their formal caregivers. The Ohmni robot was well-appreciated by all users. Informal caregivers were glad to see how easy the interface was for monitoring the Ohmni robot (SUS = 81), and believed it was very useful.

### 4.2. Phase 2 Results

In Table 8 there is a general overview of the participants recruited in Phase 2.

As in Phase 1, the distribution between the category of users (i.e., OA, IC, or FC) is not statistically significant between gender, digital skills, and educational level. On the other hand, it could be expected that the distribution of age was not the same across the users (*p* < 0.0001).

#### 4.2.1. Usability Results

Table 9 reports the SUS results of Phase 2 of the pre-validation. Regarding health management and monitoring, the SUS score of the older adults was lower than the score reported by the caregivers, similar to the socialization and inclusion support scenarios. The technologies were pre-validated in divided scenarios, as is shown in Table 9. Considering the SENTAB technology, we obtained borderline results that were comparable to the results obtained during Phase 1. The results could be explained by the very low digital skills that affected the use of new technology, and also by the stress related to the use of the technology that was measured at the beginning of the test. The scenario with the Vanilla application was pre-validated with formal and informal caregivers showed good usability, confirming the results obtained during Phase 1. The monitoring scenario with the Discovery Dashboard was appreciated by the caregivers. The Ohmni robot received comparable results to those obtained during Phase 1. Despite these low values, the feedback collected by analyzing the diaries was very positive. The Ohmni robot was the tool that impressed the most. The perception of Older Adults was that it was a “desirable” tool. It was considered non-invasive and not bulky. All of the participants were positively impressed by its ease of use.

In the case of SENTAB, people who were familiar with the use of other social networks and messenger applications considered it easier to use for video calls in particular. In addition, performing new activities such as sharing photos and texts (especially in the case of older adults) was appreciated. The Discovery Dashboard, a dedicated caregiver’s dashboard, was very appreciated. Caregivers found it very interesting and useful for monitoring the patients.

#### 4.2.2. Acceptance Results

The reliability analysis results of the AMQ test were obtained using Cronbach’s alpha analysis and are reported in Appendix A, Table A4. Regarding the deleted items, it is important to highlight that the answer to Item 23, “*I think I can use the Pharaon System without any help,*” was always not true, because the older adults all agreed that they needed some help. In any case, help from a caregiver was needed. Therefore, there is no variability in the answer. The answer for Item 25, “*I think I can use the Pharaon System when I have a good manual,*” was always not true for the same reason. In the case of Item 13, “*I think the Pharaon System can be adaptive to what I need*” the answer could be related to some malfunctioning faced during the experimental process.

**Figure 8:**
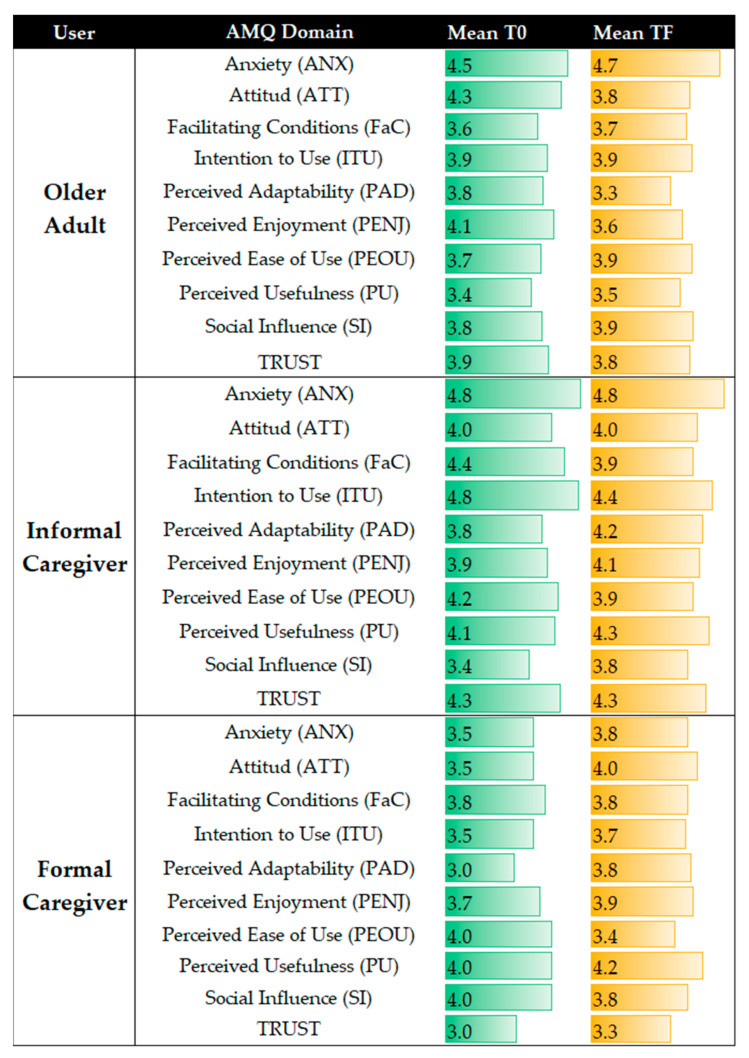
Differences between the averages of the AMQ results in T0 and in TF, divided by type of user and domain. The samples consist of six older adults, four informal caregivers, and two formal caregivers at T0; and seven older adults, five informal caregivers, and two formal caregivers at TF.

There are no differences among distribution when comparing means between T0 and TF, indicating that, in one month, there was no variability in acceptability among the population in all of the AMQ’s domains. The mean results are presented in Figure 8, divided per user. It is worth remembering that the anxiety domain had a reverse score, so a very high score is referred to as very low anxiety.

#### 4.2.3. Evaluation of Training

The reliability analysis results of the TEI are reported in Table A5 in Appendix A. The training was appreciated by the older adults, but, as was reported in AMQ results’ comments, this could be a key point in the continuous coaching provided by the caregivers. The aim was to overcome the older adults’ low digital skills; for this reason, it is clear that it was very important to train the informal caregiver. The average of the results of the test among the recruited population recruited is shown in Table 10. The answers did not differ statistically significantly among the population.

#### 4.2.4. User Experience Evaluation

The User Experience Questionnaire (UEQ) was used to assess the users’ feedback on the Pharaon system [27,46]. It was computed considering the overall population. The mean results are shown in Table 11. Cronbach’s alpha coefficients are available in Appendix A, Table A6.

Comparing our results with the benchmark intervals for the UEQ scale [46], they show that the categories of attractiveness, perspicuity, stimulation, and novelty domains reported for the Pharaon system have an excellent score. In addition, the efficiency and dependability domains reported for the Pharaon system have a good score.

#### 4.2.5. Correlation Analysis

The Pearson and Spearman correlations were calculated to determine the relationship between different tests and test domains or demographic characteristics.

Appendix A, Table A7 and Table A8, report the full correlation scores. The absence of correlations was not reported in the paper. In all tests performed, a high score is associated with a better response, except for the anxiety domain and the Technostress test. For these, we see nearly all positive correlations, excepted for the Technostress.

As was previously shown, there is a strong, positive correlation (two-tailed) between the SUS test performed during the socialization and stimulation scenario and all the domains of UEQ test. It is possible to assume that the user experience with technology affects future usability. In particular, a very strong correlation was shown in the perspicuity, dependability, and stimulation domains. However, there was a moderately positive association between the SUS test and the TEI domains. The goodness of the training did not relate strongly with the usability (Table 7).

The relationship within the AMQ domains is interesting with respect to trust and anxiety. There is a strong correlation between the anxiety domain and the perceived difficulty (PD), subjective knowledge gain (SKG), and the attitude towards training (ATT) domains. This indicates that a low level of anxiety is associated with the users’ own perceived abilities in using the technologies. In contrast, the users’ trust in technology is associated with the subjective enjoyment (SE) and perceived usefulness (PU). Confirming this, anxiety is associated with all user experience domains except attractiveness, which is strong associated with trust. In addition, trust strongly relates to dependability, efficiency, and stimulation. Therefore, it seems that trust and anxiety domains relate to different aspects of the users’ experiences and acceptances.

Stimulation and dependability are strongly associated with users’ intention To use (ITU), perceived enjoyment (PENJ), adaptability (PAD), and facilitated conditions (FaC). Moreover, the facilitated conditions are strongly associated with almost all users’ experience domains and are also very strongly associated with the attitude towards training (ATT), subjective enjoyment (SE), and stimulation domain (Table 7).

Therefore, there is an interesting, strongly negative correlation between the Technostress questionnaire performed at T0 and the mean of the perceived usefulness (PU), perceived difficulty (PD), and subjective knowledge gain (SKG) domains of the TEI questionnaire and SUS test performed during the socialization and stimulation scenario. The negative correlation exists due to the higher the score of the Technostress questionnaire and the lower digital skills of the participants, as well as their lower educational levels.

Additionally, there is a moderate, negative correlation between the age of the users and the SUS performed at T0 for the stimulation and socialization scenario (r_p_ = −0.508, n = 25, *p* = 0.010) and a strong correlation with the SUS performed at T0 for the monitoring scenario (r_p_ = −0.743, n = 9, *p* = 0.022).

### 4.3. Technology Reliability

Although some input technologies used are commercial, have high technological readiness levels (TRL), and were already validated in an operational (real user) environment, additional features, customizations, and interoperability modifications performed within the scope of the Pharaon project resulted in systems that were not yet fully validated in the real user environment and which, as expected, had new bugs that were not easy (or at all possible) to detect in the lab environment. This was one of the largest values of pre-validation from a technical perspective.

Overall, there were a total of 51 issues reported in the diaries during pre-validation in Phase 1 and Phase 2. These included 23 issues classified as major bugs, 18 minor bugs, 5 not-a-bug issues, and 5 other issues that were not classified. The issues were classified as major if the bug affected major functionality from an end user perspective and there was no workaround. Minor bugs were bugs that affected minor functionality or non-critical data for which a reasonable workaround existed (or when a workaround was not needed). Some of the issues reported were feature proposals instead of bugs. In the following months, almost all the reported issues have been addressed (45 out of 51) and partners are working to resolve the remaining few.

### 4.4. Reflection: Lessons Learned from Phase 2

The participants remarked on the fact that it is important to pre-test each new functionality before testing it in real environments, thus assessing the reliability of a certain technology as it is strictly connected to usability and acceptability. The facilitators noticed that trust in the technology (and services) decreased if older adults experienced bugs and failure (this was also confirmed by quantitative results). Another important aspect noticed by the facilitators was related to the training material. Indeed, it was not used by older adults. They preferred to call the caregiver if they had problems, believing that they could not act without the support of an external facilitator. Therefore, a specific training session for facilitators should be performed so that they could actively support the experiences of the older adults. This experience underlined that it is important to identify within each pilot a “facilitator” who can support the older adults during deployment (e.g., in Tuscany, formal and informal caregivers; in Apulia, informal caregivers would be the optimal choice). After that, the informed consent can be delivered and signed by the patients. Subsequently, the administration of baseline questionnaires and tests can be undertaken. The participants also underlined that it would be good to follow a positive attitude during the recruitment. It would be also good if the recruited older adults already knew the professional team. Another important remark was that the users would be happy to take part in the pre-validation, but they could be scared by the technology due to feelings of inadequacy. It is also good to note that the involved users decided to continue to test the services for the long-term twelve-month trial (in Apulia, four out of five older adults, four out of four and two out of two of the informal and formal caregivers, respectively; in Tuscany, five out of five older adults, five out of five and five out of five of the informal and formal caregivers, respectively) because, despite the problems, the participants enjoyed the services. Consequently, the formal/informal caregivers would be happy and assured that the patients could be appropriate users for the pre-validation. Reflection meeting participants remarked on the importance of keeping the user engaged in Phase 2; they made use of the methodology described in [50,51] to guarantee the engagement of the participants and to stimulate their use of technology over time. For example, a notification system proved to be very efficient in reaching this goal. Therefore, we decided to also apply it during the long-term deployment phase.

## 5. Discussion

The purpose of the pre-validation was to assess usability and technological reliability prior to the long-term deployment. This experience allowed the Italian pilot members to be more aware of the different aspects of adapting the methodology and the technology, if necessary. In this paper, we assessed the level of usability, acceptability, user experience, training, and stress related to the use of the technology, and we obtained quite acceptable remarks. We also investigated some RQs that direct attention to some links between domains. Thanks to the SUS results collected during Phase 1, including the qualitative feedback, the services were reshaped accordingly. For instance, the preliminary usability test underlined the low usability of the Discovery Dashboard (Table 7); therefore, the scenario was modified before long-term deployment. Additionally, thanks to the collected feedback, we decided to change the smartwatch, as it was not usable for older adults. The experience with SENTAB was good, despite its borderline usability values, so this service was not modified. The robot was highly appreciated by all participants, as was confirmed by SUS results that were high for all participants (Table 7 and Table 9). These results also align with our previous study conducted during the COVID-19 emergency [52]. Consequently, one important remark on this study concerns the discrepancies between the SUS values and the qualitative feedback collected at the end of the trial. Especially during the first phase, we obtained low SUS values even if older adults and caregivers gave facilitators quite positive feedback. As admitted by Bangor et al. [36], the SUS score seems to decrease with an increase of the age: these results have been confirmed by the strong negative-correlation analysis between the ages present in our study. One possible explanation could be that the older adults did not fully understand the items of the SUS (even the simplified version), so it is important to collect also qualitative feedback, as was remarked by [48]. The SUS tool has been defined as technology-agnostic, suitable for all type of user interfaces, and Hyzy at al. [53] demonstrated that the use of the SUS could be reliable in case of mHealth solutions. A simplified version of the SUS was proposed by Holden [54] for cognitively impaired and older adults that could not understand all the SUS statements properly. However, a new version of this scale should be developed in collaboration with the organization of end-users, ensuring that the items are rephrased understandably.

The RQ2 investigated the impact of an adequate training session on the use of the technology. The qualitative and quantitative feedback underlined that the presence of a person who can answer doubts is important in promoting the use of technology, as was also remarked during the reflection meeting. In this sense, the informal caregiver has a key role in providing continuous training on the use of technology and also reducing anxiety, among other issues. In the Tuscany pilot, older adults saw the caregiver on a weekly base, so caregivers had the opportunity to (re)train the older adults and solve bugs. Counting on the human support element behind the social network is very important as technology is not sufficient alone, and some sort of activation is required to make OAs more comfortable with the usage of technology while also providing further engagement. This could move in the direction of having both the caregiver and the technology in the loop of assistance as they can complete each other by exploiting complementary tasks. As was reported in the results, a positive correlation was present between the attitude that the users had towards training sessions and their anxiety levels. The recruitment methodology was of crucial importance and had to be focused on the heterogeneity of target users. Additionally, it is important to underline that training plays a main role in the recruitment process: it not only to shows how the technology should be used, but also engages the informal caregivers, who can guarantee continuous coaching, secured by the different environments where the technology is used [55]. Moreover, understanding the end users’ digital background knowledge was very important. In many cases, older adults were able to use devices such as smartphones; however, others were not, so the different experiences could lead to different reactions to the service. For instance, one user with high digital skills grew bored with the Sentab application installed on their tablet because they could not communicate with all of their friends; and the app was too simple for their purposes. Consequently, it is important to find a way to train the end users properly, as was also noted during the reflection session.

Regarding the RQ3, the quantitative comparison conducted at the end of Phase 2 verified that a high level of stress related to the technology could affect the perception of the technology. This was also confirmed by the qualitative feedback collected during the reflection meeting. In particular, it affected the users’ perceptions of the difficulty of using the technologies. This was probably related to digital skills and not linked to a type of user. Indeed, the stress was equally distributed among the user categories. However, in this study, we can not properly compare Technostress results with digital skills or other results, such as the anxiety domain, because not all participants completed the tests. Future studies should also investigate the relationship between stress, technology, and anxiety.

## 6. Conclusions

This paper presents the experience collected by the Pharaon consortium in Italian pilots during the two-phase pre-validation. A total of 27 persons were enrolled in Phase 1 and 27 persons in Phase 2 of this study. The proposed two-phase methodology was an important framework for minimizing risks and reducing the factors that could influence the use and acceptance of the services in prolonged trials. In addition to qualitative results, we drafted guidelines that can be used and adapted from other scholars to prepare for long-term trials. Particularly, we find three pillars that should be included in similar studies. First, we underline the important role of caregivers as the mediators between technology and older adults. Indeed, older adults experience higher stress related to technology; thus, the caregivers can facilitate training so as to include technology as a tool in the care chain. In this sense, informal caregivers can play a pivotal role, acting as mediators between the older adults and the technology. Additionally, from a future studies perspective, it is important to control the training, among other issues, by promoting tailored actions to limit the digital divide, reducing the stress related to technology. Caregivers are important for technology use and adoption; technology developers should try to understand a better way to integrate this in their work, At the same time, some more technologically oriented courses should be included into highly educational program for social/clinical operators. It is also important to have a system that promotes easy use in which older adults can trust, so to not affect the usability.

## Figures and Tables

**Table 1:** Health management and monitoring scenarios.

Addressed Needs	Technology Used	Implemented Functionality
Monitoring the environment and the older adult’s habits.	Wearable sensors (e.g., smartwatch); telepresence robot; environmental sensors; IoTool	- Measures environmental parameters (light, temperature, and humidity); - Detects when a window or door is open; - Measures activity for the day (steps and active movement); - Measures heart rate and calories during exercise and daily life; - Measures the quality of sleep.
Monitoring of an older adult’s lifestyle by the caregiver and/or professional support.	Discovery Dashboard; IoTool; SmartHabits	- Identifies anomalous behavior through pattern recognition; - Stores, collects and visualizes data related to a user in a unique dashboard.

**Table 2:** Socialization and stimulation services.

Addressed Needs	Technology Used	Implemented Functionality
Difficulties with socialization, loneliness, or a need for support.	SENTAB TV application and SENTAB Vanilla application	- Maintain a relationship with family and friends; - Obtain an overview of the socialization index; - Stay informed on the local news, events and important updates.
Poor cognitive conditions, diseases, or a sedentary lifestyle.	SENTAB TV application and SENTAB Vanilla application	- Select the type of exercise and monitor the time spent on the proposed activity and user satisfaction, and induce adherence to the exercise.

**Table 3:** List of technologies tested in the two pre-validation phases. Within Phase 1, the technology was tested as a standalone solution, whereas Phase 2 was focused on the evaluation of the integrated services.

Tested in Phase 1	Tested in Phase 2	
Technology	Socialization and Stimulation Service	Monitoring Service
SENTAB ^A^ (Older adult device on a tablet)	• ^2^	
SENTAB (Older adult device on a TV)	• ^1^	
SENTAB ^A^ (Caregiver web application; the Vanilla app)	•	
Environmental sensors (temperature and humidity; PIR) (Shelly sensors)		•
Smartwatch MAXhealth Band		•
Thingsboard and SmartHabits		•
Discovery Dashboard ^A^		• ^2^
Ohmni robot ^A^		• ^1^

^A^ Tested during Phase 1 and Phase 2 of the pre-validation. ^1^ Tested only in the Tuscany pilot. ^2^ Tested only in Apulia.

**Table 4:** Evaluation framework used in the two phases of the pre-validation. T0 and TF indicate the beginning and the end of Phase 2, respectively.

Evaluation Framework Used				
Domain	Questionnaire	Phase 1	Phase 2 T0	Phase 2 TF
Usability	System Usability Scale [20]	YES	YES	YES
Acceptability	Almere Model [21]	-	YES	YES
User Experience	User Experience Questionnaire [46]	-	-	YES
Training	Training Evaluation Inventory [30]	-	YES	-
Technostress	Perceived Stress related to technology, adapted from [45] and reported in Appendix A	-	YES	-

**Table 5:** Number of participants.

Phase	Older Adults	Informal Caregiver	Formal Caregiver	Total
1	10	10	7	27
2	14 *	9	7	30

* Three participants dropped out from the study.

**Table 6:** A brief overview of the population recruited in Phase 1.

	Older Adults n = 10	Informal Caregivers n = 10	Formal Caregivers n = 7	*p*-Value
Digital skills				
Valid Cases	n. 10	n. 9	n. 7	
Median [Q1–Q3]	1 [1.0–2.0]	1 [1.0–2.0]	2 [1.0–3.0]	0.487
Educational Level				
Valid Cases	n. 9	n. 9	n. 7	
Mean ± SD	2 [1.0–2.5]	2 [1.0–2.5]	2 [1.0–3.0]	0.812
Years				
Valid Cases	n. 9	n. 9	n. 7	
Mean ± SD	82.8 ± 12.0	55.0 ± 14.8	47.6 ± 7.3	**0.001**
Gender				
Valid Cases	n. 10	n. 9	n. 7	
Men/Women	2/8	2/7	1/6	0.909

The mean and SD for were used for normally distributed variables, and the median and IQR (Q1–Q3) were used for variables that were not normally distributed. Significan *p*-values were highlighted in bold.

**Table 7:** The mean of the SUS results in Phase 1 of pre-validation among the user categories.

Technology	Older Adults	Informal Caregivers	Formal Caregivers
SENTAB TV/Tablet	66.50	-	-
SENTAB Vanilla Application	63.50	85.36	77.00
Discovery Dashboard	48.00	80.94	93.57
Ohmni Robot	77.50	81.00	59.38

**Table 8:** General overview of participants in pre-validation Phase 2.

	Older Adults n. 11	Informal Caregivers n. 9	Formal Caregivers n. 7	*p*-Value
Digital skills				
Valid Cases	n. 11	n. 9	n. 7	
Median [Q1–Q3]	1.0 [1.0–1.0]	3.0 [1.0–3.0]	2.0 [1.0–3.0]	0.118
Educational Level				
Valid Cases	n. 11	n. 9	n.7	
Median [Q1–Q3]	1.0 [1.0–2.0]	2.0 [1.0–3.0]	2.0 [1.0–2.0]	0.422
Years				
Valid Cases	n. 11	n. 9	n. 6	
Mean ± SD	78.0 ± 7.4	48.7 ± 13.4	52.3 ± 8.8	**<0.0001**
Gender				
Valid Cases	n. 11	n. 9	n. 7	
Men/Women	3/8	5/4	2/5	0.384

The mean and SD for were used for normally distributed variables, and the median and IQR (Q1–Q3) were used for variables that were not normally distributed. Significan *p*-values were highlighted in bold.

**Table 9:** SUS results in Phase 2 of pre-validation. The sample for the SENTAB TV/tablet consisted of ten older adults. The sample for the Discovery Dashboard consisted of four informal caregivers and two formal caregivers. The sample for the SENTAB application consisted of nine informal caregivers and seven formal caregivers. The sample for the Ohmni robot scenario consisted of five Older Adults.

Technology	Older Adults		Informal Caregivers		Formal Caregivers	
	Mean ± SD T0	Mean ± SD TF	Mean ± SD T0	Mean ± SD TF	Mean ± SD T0	Mean ± SD TF
SENTAB TV/Tablet	62.0 ± 23.1	61.3 ± 19.3	-	-	-	-
Discovery Dashboard	-	-	89.2 ± 3.8 *	75.0 ± 13.2 *	71.3 ± 33.6 *	60.0 ± 7.1 *
SENTAB Vanilla Application	-	-	75.3 ± 16.8	72.2 ± 17.3	74.2 ± 14.4	63.8 ± 8.5
Ohmni Robot	58.8 ± 20.3 °	59.4 ± 19.6 °	-	-	-	-

° only the Tuscany pilot site; * only the Apulia pilot site. The SUS results, did not differ statistically significantly between T0 and TF.

**Table 10:** Training Evaluation Inventory (TEI 17-items) results.

Construct	Older Adults n. 22	Informal Caregivers n. 22	Formal Caregivers n. 22	*p*-Value
Subjective Enjoyment (SE)	4.17 [4.00–5.00]	4.67 [4.67–5.00]	4.50 [3.00–4.67]	0.420
Perceived Usefulness (PU)	3.63 ± 0.95	3.71 ± 0.86	3.38 ± 0.61	0.732
Perceived Difficulty (PD)	3.70 ± 0.79	3.96 ± 0.87	3.83 ± 0.66	0.853
Subjective Knowledge Gain (SKG)	3.07 ± 0.91	3.67 ± 1.10	3.39 ± 0.85	0.807
Attitude Towards Training (ATT)	3.17 [3.00–3.33]	3.33 [3.00–4.67]	3.33 [3.00–3.67]	0.857

The mean and SD for were used for normally distributed variables, and the median and IQR (Q1–Q3) were used for variables that were not normally distributed.

**Table 11:** UEQ scale results.

UEQ Construct	Older Adults n. 5	Informal Caregivers n. 4	Formal Caregivers n. 2
**Attractiveness**	2.33 ± 0.75	2.29 ± 0.67	1.67 ± 0.94
**Perspicuity**	1.90 ± 1.07	2.06 ± 0.55	1.50 ± 2.12
**Efficiency**	1.80 ± 1.27	1.75 ± 0.82	1.00 ± 1.41
**Dependability**	1.65 ± 1.32	1.81 ± 0.66	0.25 ± 1.06
**Stimulation**	1.95 ± 0.97	1.94 ± 0.66	1.13 ± 1.24
**Novelty**	2.50 [2.50–3.00]	1.63 [0.50–2.63]	1.00 [−0.25–2.25]

The mean and SD for were used for normally distributed variables, and the median and IQR (Q1–Q3) were used for variables that were not normally distributed.

## Data Availability

Not applicable.

## Appendix A

**Table A1:** Almere Model Questionnaire’s constructs and items used in Phase 2.

Construct Code	Original Items	Adapted Items
Anxiety (ANX)	1. If I should use the robot, I would be afraid to make mistakes with it	1. If I should use the Pharaon System, I would be afraid to make mistakes with it
	2. If I should use the robot, I would be afraid to break something	2. If I should use the Pharaon System, I would be afraid to break something
	3. I find the robot scary	3. I find the Pharaon System scary
	4. I find the robot intimidating	4. I find the Pharaon System intimidating
Attitude (ATT)	5. I think it’s a good idea to use the robot	5. I think it’s a good idea to use the Pharaon System
	6. The robot would make life more interesting	6. The Pharaon System would make life more interesting
	7. It’s good to make use of the robot	7. It’s good to make use of the Pharaon System
Facilitating Conditions (FaC)	8. I have everything I need to use the robot	8. I have everything I need to use the Pharaon System
	9. I know enough of the robot to make good use of it	9. I know enough of the Pharaon System to make good use of it
Intention to Use (ITU)	10. I think I’ll use the robot during the next few days	10. I think I’ll use the Pharaon System during the next few days
	11. I’m certain to use the robot during the next few days	11. I’m certain to use the Pharaon System during the next few days
	12. I plan to use the robot during the next few days	12. I plan to use the Pharaon System during the next few days
Perceived Adaptability (PAD)	13. I think the robot can be adaptive to what I need	13. I think the Pharaon System can be adaptive to what I need
	14. I think the robot will only do what I need at that particular moment	14. I think the Pharaon System will only do what I need at that particular moment
	15. I think the robot will help me when I consider it to be necessary	15. I think the Pharaon System will help me when I consider it to be necessary
Perceived Enjoyment (PENJ)	16. I enjoy the robot talking to me	16. I enjoy the Pharaon System talking to me
	17. I enjoy doing things with the robot	17. I enjoy doing things with the Pharaon System
	18. I find the robot enjoyable	18. I find the Pharaon System enjoyable
	19. I find the robot fascinating	19. I find the Pharaon System fascinating
Perceived Ease of Use (PEOU)	20. I find the robot easy to use	20. I find the Pharaon System easy to use
	21. I think I can use the robot without any help	21. I think I can use the Pharaon System without any help
	23. I think I can use the robot when there is someone around to help me	23. I think I can use the Pharaon System when there is someone around to help me
	22. I think I can use the robot when I have a good manual	22. I think I can use the Pharaon System when I have a good manual
Perceived Usefulness (PU)	28. I think the robot is useful to me	28. I think the Pharaon System is useful to me
	29. It would be convenient for me to have the robot	29. It would be convenient for me to have the Pharaon System
	30. I think the robot can help me with many things	30. I think the Pharaon System can help me with many things
Social Influence (SI)	31. I think the staff would like me using the robot	31. I think the staff would like me using the Pharaon System
	32. I think it would give a good impression if I should use the robot	32. I think it would give a good impression if I should use the Pharaon System
TRUST	38. I would trust the robot if it gave me advice	38. I would trust the Pharaon System if it gave me advice
	39. I would follow the advice the robot gives me	39. I would follow the advice the Pharaon System gives me

**Table A2:** Original PSS test questions and adapted PSS test questions.

The Perceived Stress Scale adapted	
Original PSS’ questions	Adapted Items in Technostress
l. In the last month, how often have you been upset because of something that happened unexpectedly?	l. How often have you been upset because of something that happened unexpectedly while using the Pharaon System?
2. In the last month, how often have you felt that you were unable to control the important things in your life?	2. How often have you felt that you were unable to control the important things in your life while using the Pharaon System?
3. In the last month, how often have you felt nervous and stressed?	3. How often have you felt nervous and stressed while using the Pharaon System?
4. In the last month, how often have you felt confident about your ability to handle your personal problems?	4. How often have you felt confident about your ability to handle your personal problems by using the Pharaon System?
5. In the last month, how often have you felt that things were going your way?	5. How often have you felt that things were going your way while using the Pharaon System?
6. In the last month, how often have you found that you could not cope with all the things that you had to do?	6. How often have you found that you could not cope with all the things that you had to do in the use of the Pharaon System?
7. In the last month, how often have you been able to control irritations in your life?	7. How often have you been able to control irritations in using the Pharaon System?
8. In the last month, how often have you felt that you were on top of things?	8. How often have you felt that you were on top of things in using the Pharaon System?
9. In the last month, how often have you been angered because of things that happened that were outside of your control?	9. How often have you been angered because of things that happened that were outside of your control in using the Pharaon System?
10. In the last month, how often have you felt difficulties were piling up so high that you could not overcome them?	10. How often have you felt difficulties were piling up so high in using the Pharaon System that you could not overcome them?

**Table A3:** The first seventeen items scales of the TEI in English and Italian.

Training Outcome Dimensions			
English		Italian	
Subjective enjoyment	Overall, I liked the training.	**Gradimento soggettivo**	Dopotutto, ho apprezzato la formazione.
	The learning atmosphere was agreeable.		L’atmosfera di apprendimento è stata gradevole.
	The learning was fun.		L’apprendimento è stato divertente.
Perceived usefulness	I find the training useful for my job (*or beyond the Pharaon project*).	**Utilità percepita**	Ho trovato la formazione utile per proseguire nella sperimentazione.
	Investing time in this training was useful.		Investire il mio tempo in questa formazione è stato utile.
	I can apply the content of this training in my job (*or beyond the Pharaon project*).		Posso applicare il contenuto di questa formazione al di fuori del progetto Pharaon.
	I derive personal use from this training (*or beyond the Pharaon project*).		Ne ho derivato un utilizzo personale al di fuori del progetto Pharaon.
Perceived difficulty	The contents were comprehensible.	**Difficoltà percepita**	Il contenuto era comprensibile.
	The language (foreign words and technical terms) was comprehensible.		Il linguaggio (termini tecnici e parole nuove) era comprensibile.
	I kept up thematically in training.		Ho continuato ad esercitarmi nel loro utilizzo dopo il training.
	The time was sufficient for the themes covered.		Il tempo di formazione è stato sufficiente per i temi affrontati.
Subjective knowledge gain	I have the impression that my knowledge has expanded on a long-term basis.	**Percezione delle competenze acquisite**	Ho l’impressione di aver acquisito delle competenze a lungo termine.
	I will be able to remember the new themes well.		Sono in grado di ricordare bene i temi.
	I think that I will still be able to report what I learned some time after the training.		Penso di essere in grado di ripetere ciò che ho imparato durante la formazione.
Attitude towards training	I will apply what I learned to my day-to-day work (or in my everyday life).	**Attitudine nella formazione**	Applicherò ciò che ho imparato nel mio nella vita di tutti i giorni.
	I find it good that data privacy was imparted and/or discussed.		Trovo importante che si sia discusso a proposito della sicurezza dei dati.
	I would recommend this training to my colleagues.		Raccomanderò la formazione ad altre persone.

**Table A4:** Results of the Cronbach’s alpha analysis.

Construct	Cronbach’s α	Item Deleted	Cronbach’s α Whit Item Deleted
Anxiety (ANX)	0.683	Item 1	0.889
Attitude (ATT)	0.641	-	-
Facilitating Conditions (FaC)	0.825	-	-
Intention to Use (ITU)	0.944	-	-
Perceived Adaptability (PAD)	0.500	Item 13	0.667
Perceived Enjoyment (PENJ)	0.748	-	-
Perceived Ease of Use (PEOU)	0.221	Item 23 Item 25	0.690 1.000
Perceived Usefulness (PU)	0.864	-	-
Social Influence (SI)	0.689	-	-
Trust	0.740	-	-

**Table A5:** The Cronbach’s alpha reliability of the Training Evaluation Inventory (TEI, seventeen items), used at the beginning (T0), compared with Cronbach’s alpha based on standardized items.

Construct	Cronbach’s α	Cronbach’s α Based on Standardized Items
Subjective Enjoyment (SE)	0.851	0.864
Perceived Usefulness (PU)	0.809	0.817
Perceived Difficulty (PD)	0.814	0.827
Subjective Knowledge Gain (SKG)	0.822	0.829
Attitude Towards Training (ATT)	0.698	0.701

**Table A6:** The Cronbach’s alpha reliability for the UEQ’s domains.

Construct	Cronbach’s α
**Attractiveness**	0.844
**Perspicuity**	0.820
**Efficiency**	0.864
**Dependability**	0.792
**Stimulation**	0.851
**Novelty**	0.940

**Table A7:** Correlation scores.

Hypothesis	Independent Variables	Dependent Variable	The Number of Participants Included	Correlation	Sig. (2-Tailed)
RQ1	FaC	SUS Socialization and Stimulation scenario	11	**0.896 (R_S_)**	0.0002
	ITU		11	**0.653 (R_P_)**	0.029
	Trust		11	**0.611 (R_P_)**	0.045
	Attractiveness		11	**0.738 (R_P_)**	0.010
	Perspicuity		11	**0.845 (R_P_)**	0.001
	Efficiency		11	**0.665 (R_P_)**	0.026
	Dependability		11	**0.824 (R_P_)**	0.002
	Stimulation		11	**0.846 (R_P_)**	0.001
	Novelty		11	**0.647 (R_S_)**	0.031
RQ2	PU of TEI	SUS Socialization and Stimulation scenario	21	**0.641 (R_P_)**	0.002
	PD of TEI		21	0.552 (R_P_)	0.009
	SKG of TEI		21	0.472 (R_P_)	0.031
	ATT of TEI		21	0.578 (R_S_)	0.006
	FaC of AMQ	SE of TEI	11	**0.826 (R_S_)**	0.002
	ITU of AMQ		11	**0.708 (R_P_)**	0.015
	Trust of AMQ		11	**0.648 (R_P_)**	0.031
	ATT of AMQ	PU of TEI	11	**0.770 (R_P_)**	0.006
	Trust of AMQ		11	**0.748 (R_P_)**	0.008
	ANX of AMQ	PD of TEI	11	**0.619 (R_P_)**	0.042
	ANX of AMQ	SKG of TEI	11	**0.618 (R_P_)**	0.043
	Trust of AMQ		11	**0.623 (R_P_)**	0.041
	FaC of AMQ		11	**0.712 (R_S_)**	0.014
	ANX of AMQ	ATT of TEI	11	**0.762 (R_S_)**	0.006
RQ2	FaC of AMQ		11	**0.903 (R_S_)**	0.0001
	PENJ of AMQ		11	**0.694 (R_S_)**	0.018
	ITU of AMQ	Attractiveness of UEQ	11	**0.641 (R_P_)**	0.040
	PENJ of AMQ		11	**0.641(R_P_)**	0.033
	Trust of AMQ		11	**0.643 (R_P_)**	0.033
	FaC of AMQ		11	**0.745 (R_S_)**	0.009
	ANX of AMQ	Perspicuity of UEQ	11	**0.639 (R_P_)**	0.034
	FaC of AMQ		11	**0.741 (R_S_)**	0.009
	ANX of AMQ	Efficiency of UEQ	11	**0.635 (R_P_)**	0.036
	PAD of AMQ		11	**0.779 (R_P_)**	0.005
	Trust of AMQ		11	**0.653 (R_P_)**	0.029
	FaC of AMQ		11	**0.636 (R_S_)**	0.035
	PU of AMQ		11	**0.636 (R_S_)**	0.035
	ANX of AMQ	Dependability of UEQ	11	**0.651 (R_P_)**	0.030
	ATT of AMQ		11	**0.691 (R_P_)**	0.019
	ITU of AMQ		11	**0.667 (R_P_)**	0.025
	PAD of AMQ		11	**0.676 (R_P_)**	0.022
	PENJ of AMQ		11	**0.617 (R_P_)**	0.043
	Trust of AMQ		11	**0.821 (R_P_)**	0.002
	FaC of AMQ		11	**0.772 (R_S_)**	0.005
	ANX of AMQ	Stimulation of UEQ	11	**0.756 (R_P_)**	0.007
	ITU of AMQ		11	**0.617 (R_P_)**	0.043
	PAD of AMQ		11	**0.703 (R_P_)**	0.016
	PENJ of AMQ		11	**0.699 (R_P_)**	0.017
	Trust ofAMQ		11	**0.686 (R_P_)**	0.020
	FaC of AMQ		11	**0.893 (R_S_)**	0.0002
	PU of AMQ		11	**0.670 (R_S_)**	0.024
	ANX of AMQ	Novelty of UEQ	11	**0.695 (R_S_)**	0.018
	ATT of AMQ		11	**0.772 (R_S_)**	0.005

***Legend***: R_P_: Pearson coefficient; R_S_: Spearman coefficient; System Usability Scale (SUS); Training Evaluation Inventory (TEI) domains: Subjective Enjoyment (SE); Perceived Usefulness (PU); Perceived Difficulty (PD); Subjective Knowledge Gain (SKG); Attitude Towards Training (ATT); Almere Model Questionnaire (AMQ) domains: Anxiety (ANX); Attitude (ATT), Facilitating Conditions (FaC), Intention to Use (ITU), Perceived Adaptability (PAD), Perceived Enjoyment (PENJ), Perceived Usefulness (PU), Social Influence (SI), Trust; User Experience Questionnaire (UEQ). Bold correlation values are the sinificant ones.

**Table A8:** Correlation scores. R_p_ represents the Pearson Correlation Coefficient and R_s_ represents the Spearman correlation coefficient.

Hypothesis	Independent Variables	Dependent Variable	The Number of Participants Included	Correlation	Sig. (2-Tailed)
RQ3	SUS_S_T0	Technostress (T0)	16	**−0.648 (R_P_)**	0.007
	PU of TEI		15	−0.576 (R_P_)	0.025
	PD of TEI		15	**−0.618 (R_P_)**	0.014
	SKG of TEI		15	**−0.686 (R_P_)**	0.005
	FaC of AMQ		5 *	**−0.949 (R_S_)**	0.014
	Perspicuity		5 *	**−0.898 (R_P_)**	0.040
	Efficiency		5 *	**−0.888 (R_P_)**	0.044
	Stimulation		5 *	**−0.906 (R_p_)**	0.034

* a very strong negative correlation but very small sample. Bold correlation values are the sinificant ones. ***Legend***: R_P_: Pearson coefficient; R_S_: Spearman coefficient; System Usability Scale (SUS); Training Evaluation Inventory (TEI) domains: Perceived Usefulness (PU); Perceived Difficulty (PD); Subjective Knowledge Gain (SKG); Almere Model Questionnaire (AMQ) domains: Facilitating Conditions (FaC), User Experience Questionnaire (UEQ).
